# An automated deep learning-based pipeline for 3D characterisation of the murine upper airway from micro-CT

**DOI:** 10.3389/fmedt.2026.1823048

**Published:** 2026-08-11

**Authors:** Flore Belmans, Sergi Llambrich, Wim Vos, Frederik Maes, Neus Martínez-Abadías, Greetje Vande Velde

**Affiliations:** 1 Department of Imaging and Pathology, KU Leuven, Leuven, Belgium; 2 Radiomics.bio, Liège, Belgium; 3 Departament de Biologia Evolutiva, Ecologia i Ciències Ambientals (BEECA), Facultat de Biologia, Universitat de Barcelona (UB), Barcelona, Spain; 4 Department ESAT/PSI, KU Leuven, Leuven, Belgium; 5 Medical Imaging Research Center, UZ Leuven, Leuven, Belgium

**Keywords:** convolutional neural networks, imaging biomarkers, lung disease, micro-computed tomography, respiratory tract, segmentation, small animal models

## Abstract

**Introduction:**

Upper airway structure and function are affected in several respiratory (e.g., obstructive sleep apnoea, allergic rhinitis, and asthma) and congenital [e.g., Down syndrome (DS)] conditions. Although murine models are widely used to study airway pathology and to evaluate therapies, quantitative analysis of the upper airway on micro-computed tomography (micro-CT) remains limited as it is hampered by labour-intensive and observer-dependent manual segmentation of the images. Automated and standardized image analysis methods are therefore needed to support mechanistic and translational upper airway research.

**Materials and methods:**

We developed a deep learning-based pipeline for fully automated segmentation and characterization of the trachea, pharynx, and nasal cavity from micro-CT images. A full-resolution 2D CNN based on the nnU-Net framework was trained in the axial plane using 5-fold cross-validation on 106 scans from a DS study involving wild-type (WT) and trisomic (TS) mice. Generalisability was assessed on an external validation set of 74 scans from another DS study examining the effect of RSV infection in WT and TS mice. Model performance was evaluated using the Dice similarity coefficient (DSC), relative absolute volume difference (RAVD), Pearson correlation and Bland–Altman analysis between manual and automated measurements.

**Results:**

Strong segmentation performance was obtained on the internal validation set, with DSC of 0.988 ± 0.010, 0.985 ± 0.008, and 0.958 ± 0.004, and RAVD of 1.483% ± 1.800%, 1.506% ± 1.217% and 1.965% ± 1.563% for trachea, pharynx and nasal cavity, respectively. Performance remained robust on the external validation set, confirming generalisability across different experimental conditions and imaging protocols. Automatically extracted volumetric biomarkers showed strong agreement with manual measurements and small Bland–Altman biases. The pipeline reproduced biologically relevant group differences previously identified through manual analysis, including reduced pharynx and nasal cavity volumes in TS mice compared with WT controls.

**Conclusions:**

We present a deep learning-based pipeline for automated, standardized 3D characterization of the murine upper airway from micro-CT. By reducing manual workload and enhancing measurement consistency, it enables high-throughput phenotyping and mechanistic investigation of the upper airway in preclinical models. By facilitating direct, automated measurements from imaging, our pipeline strengthens the translational bridge between experimental and clinical research.

## 1 Introduction

Respiratory diseases such as obstructive sleep apnoea, allergic rhinitis and asthma, as well as congenital disorders that are associated with midfacial hypoplasia, such as Down syndrome (DS) or syndromic craniosynostoses, involve structural or functional abnormalities of the upper airway, including the nasal cavity, pharynx, and trachea. Upper airway abnormalities lead to breathing difficulties, disrupted sleep, diminished quality of life, and, in severe cases, potentially life-threatening complications (1–4). An important risk factor for developing obstructive sleep apnoea is obesity, whose prevalence continues to rise globally (5). In individuals with DS, anatomical differences including a smaller trachea, increase the risk of airway obstruction (3, 6, 7). In clinical practice, evaluation of the upper airway typically combines physical examination with imaging techniques such as computed tomography, magnetic resonance imaging, ultrasound or endoscopy (8). These techniques are essential for diagnosing airway obstruction, characterizing structural variations and guiding therapeutic interventions.

To link clinical observations to mechanistic understanding and therapeutic innovation, experimental animal models are being used to study disease processes under controlled conditions and to test novel therapies before clinical application. Among these, murine models are widely used due to their low cost, easy handling, short breeding time and availability of various transgenic and knockout strains (9). Accurate assessment of the upper airway in these models is crucial in respiratory research for linking genotype to phenotype, assessing disease progression, and evaluating therapeutic response. Traditionally, the murine upper airway is studied using invasive or indirect approaches, such as *ex vivo* cell and tissue analysis, which provide limited spatial information and require terminal procedures (10, 11). The use of intravital optical imaging such as OCT or microscopy is rare for the upper airway, or limited to the trachea in specialized setups (12, 13). In contrast, micro-computed tomography (micro-CT) has emerged as a powerful, translational tool for non-invasive, longitudinal, 3D quantitative assessment of small animal anatomy and pathology, including the respiratory tract (14). Few studies have used *in vivo* micro-CT to characterize the murine upper airway, for example to investigate upper airway hyperresponsiveness in eosinophilic upper airway inflammation or respiratory deformations in a model of DS (15, 16).

Micro-CT analysis typically starts with the identification of the volume of interest (VOI), i.e., outlining the specific upper airway structures to be analysed in the 3D image, also referred to as segmentation. Quantitative measurements describing volume or tissue density can be extracted from the VOI and used as imaging biomarkers to assess disease progression or response to treatment. Additionally, 3D meshes can be computed from the VOI and can be used to quantify shape differences using geometric morphometrics, a sophisticated body of statistical tools developed for measuring and comparing shapes with increased precision and efficiency (4, 17). Finally, 3D models can be used to investigate flow and pressure profiles in the upper airway using computational fluid dynamics (CFD) (16, 18).

However, the segmentation of the VOI on micro-CT is typically performed slice-by-slice manually or semi-automatically, which is time-consuming, labour-intensive and susceptible to both inter- and intra-observer variability and 3D segmentation inconsistencies. These challenges pose a significant barrier to high-throughput studies and reproducibility across laboratories. Recent advances in artificial intelligence (AI), in particular deep learning (DL) using convolutional neural networks (CNN), have unlocked new possibilities for automating image analysis in biomedical research. Among these, the nnU-Net framework has emerged as a state-of-the-art solution for medical image segmentation (19). By automatically adapting its architecture and training pipeline to a given dataset, nnU-Net has demonstrated robust performance across a wide range of biomedical imaging tasks, without the need for manual parameter tuning. Although several methods have already been developed for the automated segmentation of the human upper airway from CT images (20), to our knowledge, no automated methods currently exist for segmenting the murine upper airway on micro-CT.

In this study, we present an automated DL-based pipeline for the 3D segmentation and quantification of the murine upper airway, including trachea, pharynx and nasal cavity, from micro-CT scans using a full-resolution 2D CNN. Additionally, we investigate the relevance of our pipeline for respiratory research by assessing its ability to detect biological differences between different experimental groups using a mouse model for Down syndrome. We show that our pipeline significantly reduces the manual workload and is able to detect biologically meaningful results, offering a scalable and reproducible solution for airway phenotyping in experimental models.

## 2 Materials and methods

### 2.1 Datasets

Micro-CT datasets of two different experimental studies were retrospectively used to develop and evaluate a DL model for upper airway segmentation on micro-CT (21, 22). An overview of the complete data-processing pipeline, from image acquisition to statistical analysis, is provided in Supplementary Figure S1. An overview of the datasets can be found in Table 1.

**Table 1:** Overview of micro-CT datasets used in this study for training/internal and external validation.

Parameter	Training and internal validation	External validation
Dataset acronym	DS-TR	DS-RSV
Disease model	Down syndrome + treatment/control	Down syndrome + RSV/dPBS
REF	(21)	(22)
Nr of animals	55	39
Male, *n* (%)	22 (40)	39 (100)
Trisomic, *n* (%)	25 (45)	20 (51)
Treated, *n* (%)	36 (65)	0 (0)
RSV infected, *n* (%)	0 (0)	24 (62)
Nr of timepoints, *n*	2 (51 mice) or 1 (4 mice)	2 (35 mice) or 1 (4 mice)
Nr of scans, *n*	106	74

All preclinical data used in this study were obtained in accordance with national and European regulations concerning animal research. The experiments were reviewed and approved by the animal ethics committee of the KU Leuven (ECD). Where no new animal data was acquired for this work, details on the approval of the original animal experiments by the ethical committee can be found in the respective publications.

The dataset used for model training and internal validation (DS-TR in Table 1) consisted of mice obtained by crossing Ts65Dn [B6EiC3Sn-a/A-Ts (1716)65Dn] females and B6EiC3Sn.BLiAF1/J males (refs. 005252 and 003647, the Jackson Laboratory Bar Harbor, ME, USA) resulting in 55 F1 trisomic (TS) and euploid wildtype (WT) littermates (21). A total of 13 litters were bred, six were left untreated, and seven were treated with green tea extracts enriched in epigallocatechin-3-gallate (GTE-EGCG, Mega Green Tea Extract, Life Extension, USA), a pharmacological compound with proven potential to modulate craniofacial development in Ts65Dn mice and potentially the upper airway (23, 24). Treatment was administered from embryonic day 9 (E9) until the mice reached 5 months of age, after which they received water until the end of the experiment at 8 months of age. The treatment was administered through maternal drinking water *ad libitum* at a concentration of 0.09 mg/mL from E9 until weaning at PD21. After weaning, the treatment was given directly to the mice at the same concentration until 5 months of age. *In vivo* micro-CT scans were performed at two timepoints: during treatment administration at 5 months of age in WT and TS treated mice, and six and a half months of age in WT and TS untreated mice; and after treatment discontinuation at 8 months of age in all mice. 4 mice were only scanned at the first timepoint because they died before the second timepoint.

The dataset for external validation (DS-RSV in Table 1) consisted of 39 6 to 7-week-old male Ts65Dn mice and euploid wildtype littermates obtained from the Jackson Laboratory (refs. 005252 and 003647, the Jackson Laboratory Bar Harbor, ME, USA) (22). The mice were randomly assigned to be either sham infected with dPBS or infected with a luciferase-recombinant human respiratory syncytial virus (rhRSV-Luc) Long strain, propagated in Hep-2 cells according to a standard procedure to obtain high-titre viral stocks (25). Mice were anesthetized using 2 mL/min isoflurane and infected intranasally with 50 μL of dPBS containing vehicle (sham) or 1.7 × 10^6^ PFU/mL rhRSV-Luc. *In vivo* micro-CT scans were performed at baseline before infection and four days after infection. 4 mice were only scanned at the first timepoint because they died before the second timepoint.

The scan parameters for the micro-CT datasets used in this study are listed in Table 2. Both datasets were acquired with a dedicated whole-body small animal micro-CT scanner (SkyScan1278, Bruker micro-CT, Kontich, Belgium). The animals were imaged in a free-breathing anesthetized state. The images were reconstructed using NRecon (NRecon software version 1.7.3.1, Bruker Micro-CT, Kontich, Belgium) adjusting the ring artifact and post-alignment correction for optimal upper airway visualization with a beam hardening correction of 10%, a smoothing of 1, and an image range attenuation coefficient of −0.003 (−0.002 for DS-RSV) and 0.05 for both datasets. The reconstructed images were then rotated using DataViewer (DataViewer software version 1.5.6.2, Bruker Micro-CT, Kontich, Belgium) to ensure consistent orientation and symmetry across all scans and facilitate manual segmentation. Each acquisition resulted in a reconstructed 3D dataset with isotropic voxel size of 51 μm.

**Table 2:** Technical scan parameters for the micro-CT datasets used in this study for training/internal and external validation.

Parameter	Training and internal validation	External validation
Dataset acronym	DS-TR	DS-RSV
Voxel size (μm)	51	51
Scanner	SkyScan1278	SkyScan1278
Source voltage (kV)	55	50
Filter	Al 1 mm	Al 1 mm
Source current (μA)	700	350
Exposure time (ms)	80	150
Step increment (°)	1	0.9
Total angle (°)	360	220
Time (min)	3	3
Frame averaging	2	4
Image matrix width, median (Q1–Q3)	602 (584–656)	528 (509–556)
Image matrix height, median (Q1–Q3)	512 (481–628)	494 (480–512)
Number of axial slices, median (Q1–Q3)	859.5 (811.25–902)	931 (892.5–988.5)

**Figure 1:**
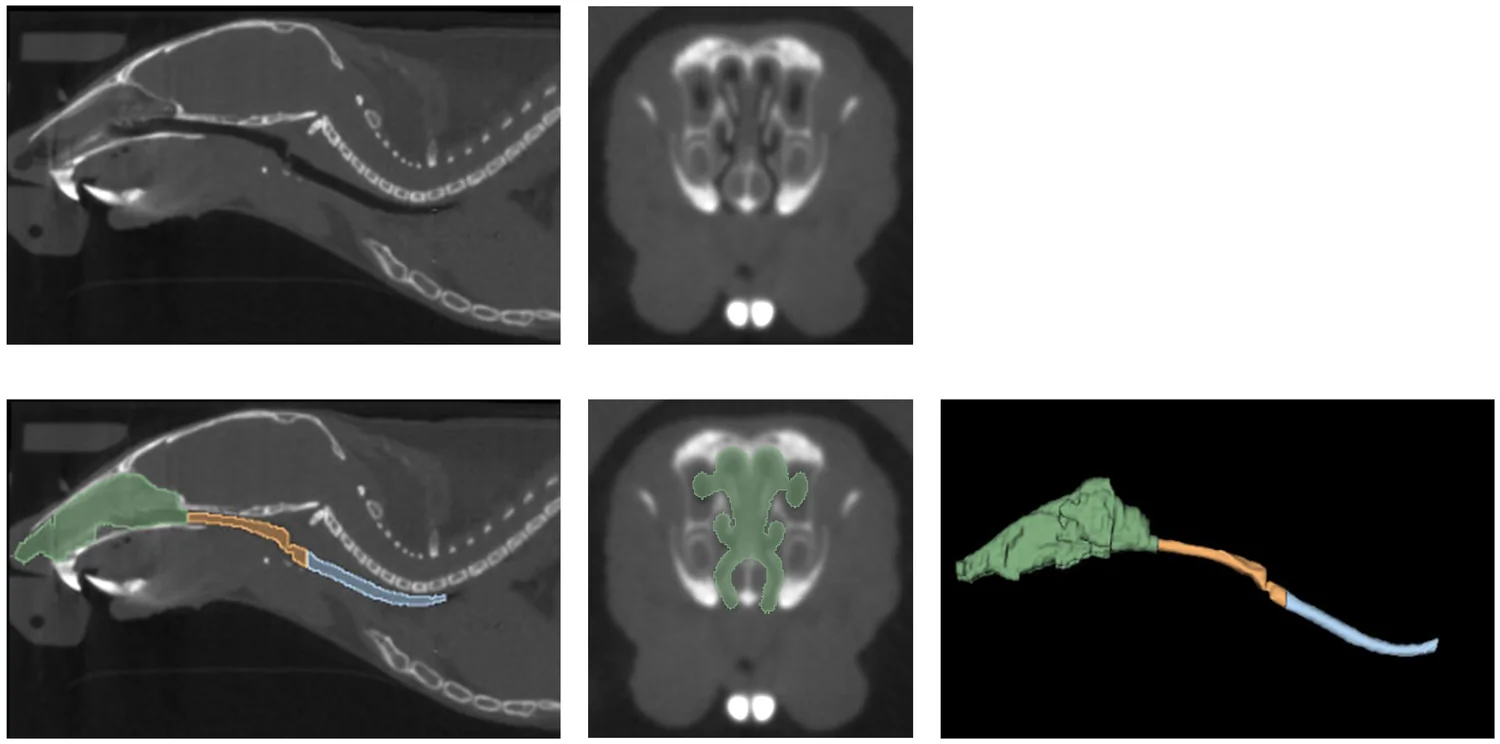
Example of a micro-CT scan with manual segmentation of the upper airway. (Upper) Sagittal and axial slice of a micro-CT scan with (lower) manual segmentation of the trachea (blue), pharynx (orange) and nasal cavity (green) overlaid and corresponding 3D visualization.

The VOI consisting of the upper airway was delineated once by one of two experienced biomedical scientists following a standardized annotation protocol based on predefined anatomical landmarks using 3D Slicer (v5.0.2; http://www.slicer.org) (Figure 1) (26). In all datasets, the trachea and pharynx were semi-automatically segmented by tuning a threshold and removing voxels to select only the aerated region from the tracheal bifurcate to the start of the tracheal rings for the trachea, and from the start of the tracheal rings to the nasopharyngeal meatus for the pharynx. For all scans in the DS-TR dataset and 10 scans in the DS-RSV dataset, the nasal cavity was completely manually segmented by delineating the aerated region and inner cartilage on each axial image slice from the nasopharyngeal meatus to the nostrils.

### 2.2 Model development

The training data, composed of 106 scans of 55 animals (Table 1), was split into 5 sets for 5-fold cross-validation (Supplementary Table S1). The split was performed on the animal level such that all longitudinal scans of one animal were included in the same fold. In addition, the split was stratified on sex, genotype and treatment to ensure a similar distribution over the different folds. The split resulted in four folds of 21 scans and one fold of 22 scans. The intensities of all scans were normalized to equalize appearance across datasets by automatically estimating Hounsfield units from the original grey values using histogram-based analysis (27).

A full-resolution 2D CNN based on the state-of-the-art nnU-Net framework (version 2.5.2) (19) was trained with 5-fold cross-validation to segment the trachea, pharynx and nasal cavity as different labels on murine micro-CT. Residual connections were added in the encoder of the U-Net based architecture using the “ResEnc M” preset (28). The framework automatically adapted the architecture to our training dataset resulting in an 8-stage architecture with [32, 64, 128, 256, 512, 512, 512, 512] feature maps per stage, a patch size of 512 × 512 taken in the axial plane and batch size of 12. The CT input was normalized by clipping intensities to the 0.5 and 99.5 percentiles of all foreground intensities computed from all training cases ([−600, 1,711]), then applying *z*-score normalization using the mean (*µ* = 106.5) and standard deviation (*σ* = 430.5) of those foreground intensities. The model weights were optimized with respect to the sum of Dice and cross-entropy loss using the stochastic gradient descent optimizer with a high initial learning rate (0.01) and a large Nesterov momentum (0.99), which was reduced during training using the “polyLR” schedule. All trainings ran for a fixed length of 1,000 epochs, as recommended (19). More details on the training scheme and data augmentation can be found in the respective nnU-Net publication (19). All models were trained on a NVIDIA GeForce RTX 2080 Ti GPU with 11 GB memory size.

Model predictions were made patch-wise using a sliding window approach, with an overlap of 256 pixels and the resulting probability maps for each label were converted into binary segmentations by thresholding at 0.5. An ensemble model was constructed by averaging the probability maps of the predictions made by the 5 models resulting from 5-fold cross-validation, before binarization. All resulting segmentations were automatically post-processed by removing small, disconnected clusters of pixels and filling small holes. To obtain a consistent starting point of the trachea, slices where the trachea split into the bronchi were removed from the segmentation. Moreover, to mimic the manual segmentation protocol, each axial slice was constrained to a single anatomical label. In the manual reference segmentations, the airway subregions were defined as consecutive anatomical segments along the cranio-caudal axis, such that each axial slice was assigned to only one region. To enforce consistency with this definition and eliminate occasional within-slice label inconsistencies at region boundaries, all segmented voxels within a slice were assigned the most prevalent label in that slice.

### 2.3 Model evaluation

Internal validation was performed by evaluating each model on its corresponding held-out fold across the 5-fold cross-validation training setup. To assess the generalizability of the models beyond the training data, external validation was performed by evaluating the ensemble model on the independent DS-RSV dataset, which was not involved in training. Upper airway segmentation performance was assessed by comparing automated segmentations of the trachea, pharynx and nasal cavity to manual reference segmentations using the Dice similarity coefficient (DSC) and relative absolute volume difference (RAVD), both widely accepted and complementary metrics for evaluating segmentation performance in medical imaging (29). DSC quantifies spatial overlap between segmentations, ranging from 0 (no overlap) to 1 (perfect overlap), while RAVD captures the relative volumetric error, particularly important for volumetric biomarker extraction, and is calculated as (|volume_predicted – volume_manual|/volume_manual) × 100%. Results are reported as mean ± standard deviation (SD), to describe variability across scans.

In the external validation dataset, manual segmentation of the nasal cavity by an experienced biomedical scientist was performed for 10 of the 74 scans due to the labour-intensive nature of the task. To maximise heterogeneity, five scans were selected from timepoint 1 and five from timepoint 2, while ensuring representation of all experimental groups. For the same 10 scans, the nasal cavity was automatically segmented using the ensemble model, and the automated segmentations were subsequently visually inspected by the same expert and manually corrected where deemed needed. These corrected segmentations were used to evaluate practical usability and required correction effort of the automated segmentations but were not considered independent ground truth for formal validation purposes. The DSC and RAVD metrics were computed between manual, automated and corrected segmentations.

### 2.4 Accuracy of biomarker quantification

Model performance was further evaluated by comparing the results of volumetric biomarker extraction from the manually and automatically segmented upper airway. Bland–Altman analysis (30, 31) was conducted to assess the agreement between manually and automatically extracted biomarkers, being trachea, pharynx and nasal cavity volume. The mean difference (bias) and 95% limits of agreement as ±1.96 standard deviations of the differences were calculated.

### 2.5 Biological relevance

The utility of the developed DL-based automated pipeline was assessed by evaluating its ability to detect biologically meaningful differences in the upper airway of murine models. In the internal validation dataset, we examined the effects of genotype and treatment on the imaging-derived biomarkers of the upper airway, while in the external validation dataset, we evaluated the effects of genotype and RSV infection on these biomarkers. A mixed-design ANOVA with time (two levels) as within-subject factor and experimental group as between-subject factor was conducted on the respective biomarkers. Animals with missing measurement at the second timepoint were excluded from the longitudinal analysis (complete-case analysis). Assumptions of normality and homogeneity of variance were assessed prior to analysis. *Post-hoc* pairwise comparisons between groups, averaged across timepoints, were performed using Tukey's test only when the main effect of experimental group reached statistical significance. We investigated whether the same statistically significant differences were retrieved with the automated pipeline vs. manual analysis. Statistical significance was set at *p* < 0.05.

## 3 Results

### 3.1 Model evaluation

The model's segmentation performance was evaluated by comparing the automated with the manual reference segmentations, using DSC to assess overlap and RAVD to assess volumetric agreement. On the internal validation set, a mean DSC of 0.988 ± 0.010, 0.985 ± 0.008 and 0.958 ± 0.004 and a mean RAVD of 1.483% ± 1.800%, 1.506% ± 1.217% and 1.965% ± 1.563% was obtained for the trachea, pharynx and nasal cavity, respectively (Table 3).

**Table 3:** Internal validation results. DSC and RAVD obtained by 5-fold cross-validation for trachea, pharynx and nasal cavity.

Fold	*n*	Trachea		Pharynx		Nasal cavity	
		DSC (−)	RAVD (%)	DSC (−)	RAVD (%)	DSC (−)	RAVD (%)
Fold 1	21	0.986 ± 0.010	1.452 ± 2.021	0.985 ± 0.006	1.674 ± 1.130	0.957 ± 0.004	2.477 ± 2.039
Fold 2	22	0.988 ± 0.006	1.593 ± 1.187	0.984 ± 0.008	1.591 ± 1.057	0.958 ± 0.003	1.941 ± 1.244
Fold 3	21	0.989 ± 0.011	1.252 ± 1.752	0.989 ± 0.007	0.784 ± 0.665	0.958 ± 0.005	1.670 ± 1.247
Fold 4	21	0.988 ± 0.008	1.311 ± 1.051	0.984 ± 0.009	2.035 ± 1.597	0.958 ± 0.003	1.993 ± 1.725
Fold 5	21	0.988 ± 0.013	1.801 ± 2.536	0.985 ± 0.009	1.441 ± 1.096	0.958 ± 0.003	1.774 ± 1.288
Total	106	0.988 ± 0.010	1.483 ± 1.800	0.985 ± 0.008	1.506 ± 1.217	0.958 ± 0.004	1.965 ± 1.563

Results are reported as mean ± standard deviation across the respective samples.

**Figure 2:**
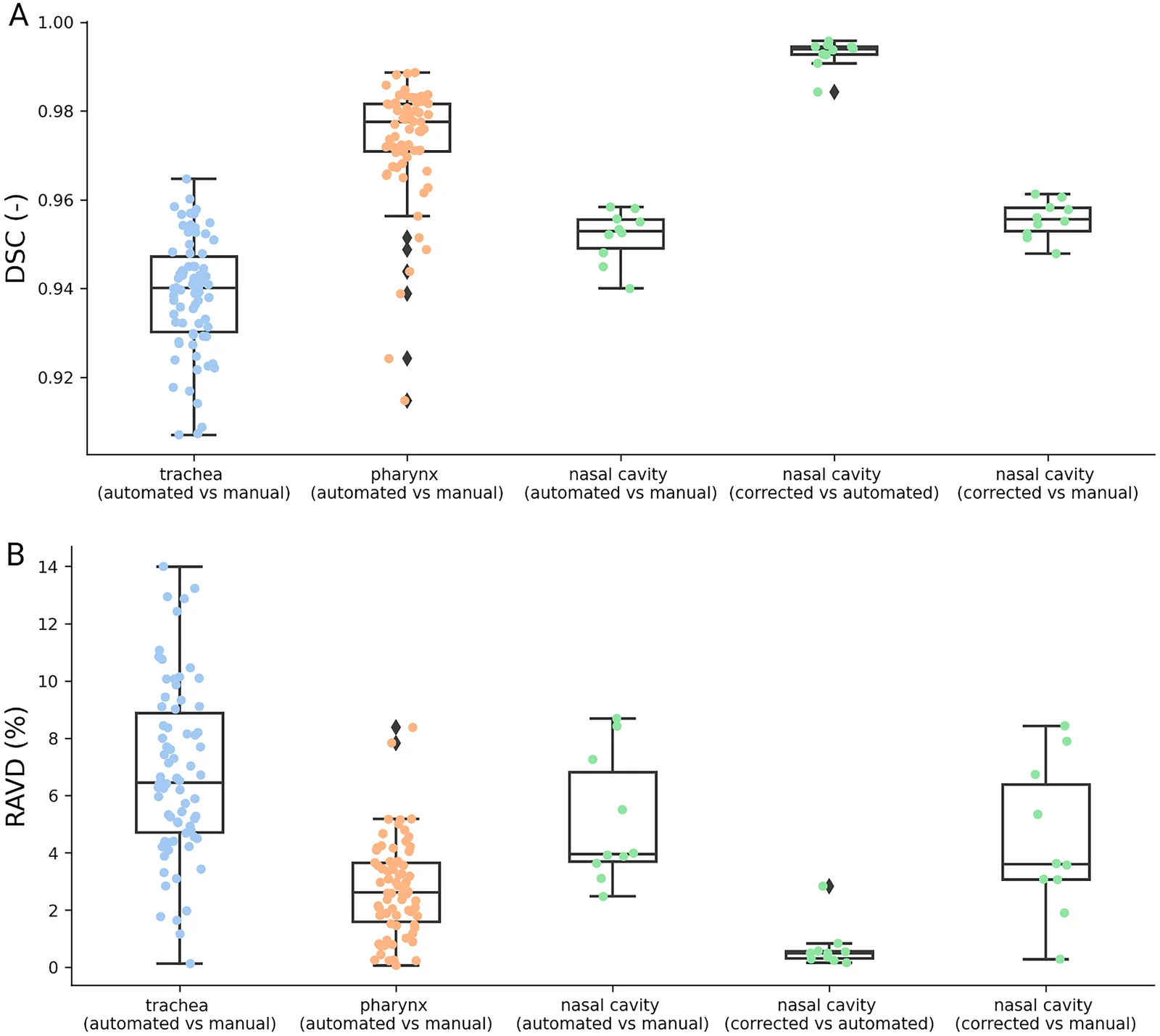
External validation results for the ensemble model. **(A)** DSC and **(B)** RAVD obtained for trachea (automated vs. manual), pharynx (automated vs. manual) and nasal cavity (automated vs. manual, corrected vs. automated, corrected vs. manual).

**Figure 3:**
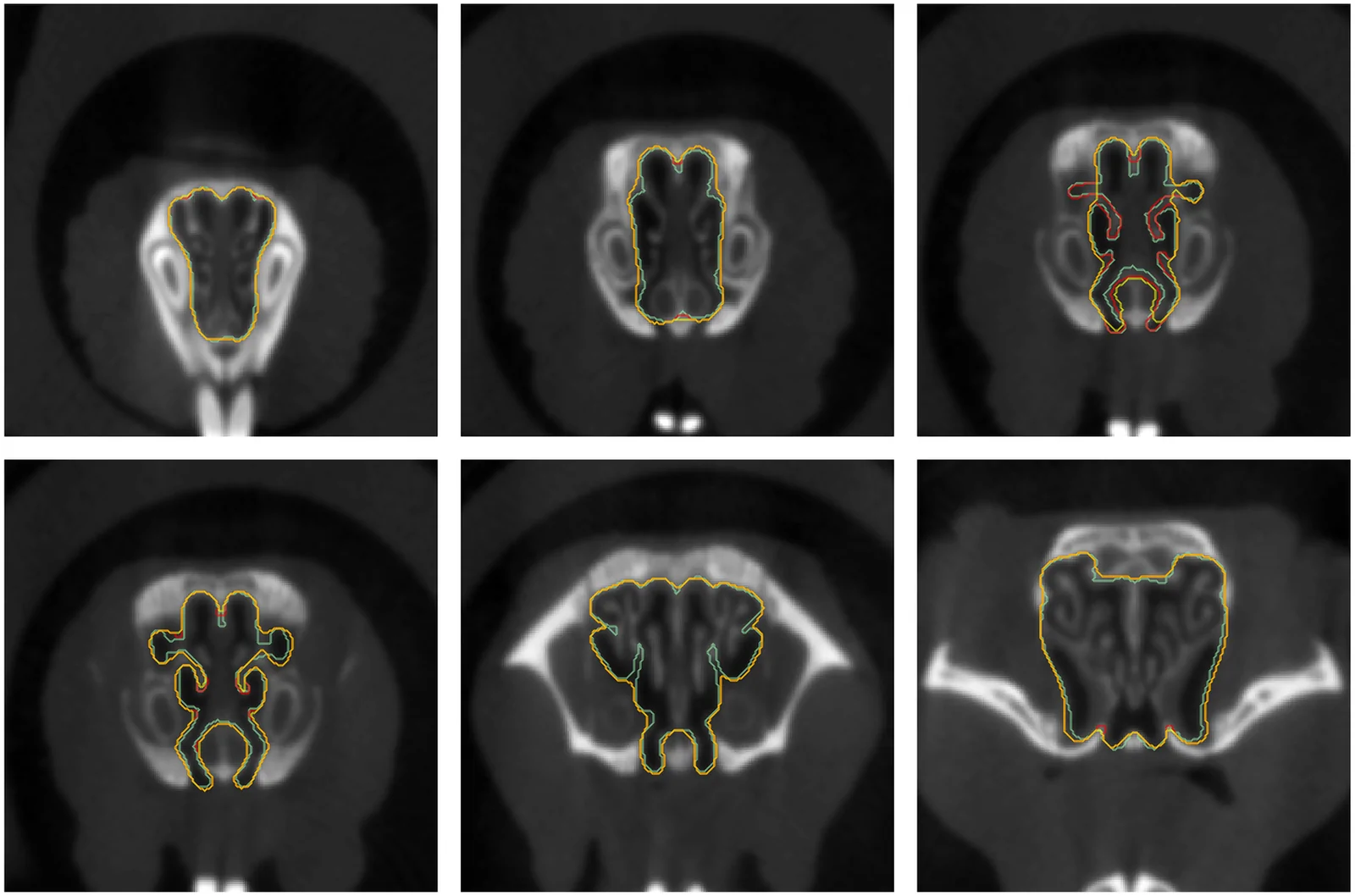
Manual, automated and corrected nasal cavity segmentations. Comparison between manual (green), automated (yellow) and corrected (red) nasal cavity segmentations for an external validation case with DSC = 0.948 between the automated and manual segmentation. Only in few slices extensive corrections of the automated segmentation were needed, while in most slices only minor corrections were made by the expert.

Next, we assessed the generalisability of the model beyond the training data by testing the ensemble model on the external validation set. The mean DSC values for the trachea, pharynx, and nasal cavity were 0.939 ± 0.013, 0.973 ± 0.013, and 0.952 ± 0.006, respectively, while the corresponding mean RAVD values were 6.799% ± 2.976%, 2.733% ± 1.634%, and 5.083% ± 2.142% (Figure 2). When comparing the automated to the corrected nasal cavity segmentations, the agreement improved to a mean DSC of 0.993 ± 0.003 and a mean RAVD of 0.678% ± 0.741% (Figure 2). When compared to the manual nasal cavity segmentations, the corrected segmentations achieved a mean DSC of 0.956 ± 0.004 and a mean RAVD of 4.389% ± 2.507% (Figure 2). Figure 3 shows a representative example with the manual, automated and corrected nasal cavity segmentations overlaid on the micro-CT. In most slices, the automated and manual segmentations closely overlapped. In slices with discrepancy between both, the expert performing corrections often followed the automated segmentation, performing no or only minimal adjustments. Only in a few slices with more complex contours, more extensive manual corrections were performed. Overall, these results show that our model generalizes well from the internal to the external validation set, with metrics remaining in the same range (DSC > 0.93, RAVD < 7%). Moreover, the automated nasal cavity segmentations required only minimal manual correction to obtain acceptable VOIs, showing the ambiguity of the manual reference standard.

### 3.2 Accuracy of biomarker quantification

**Figure 4:**
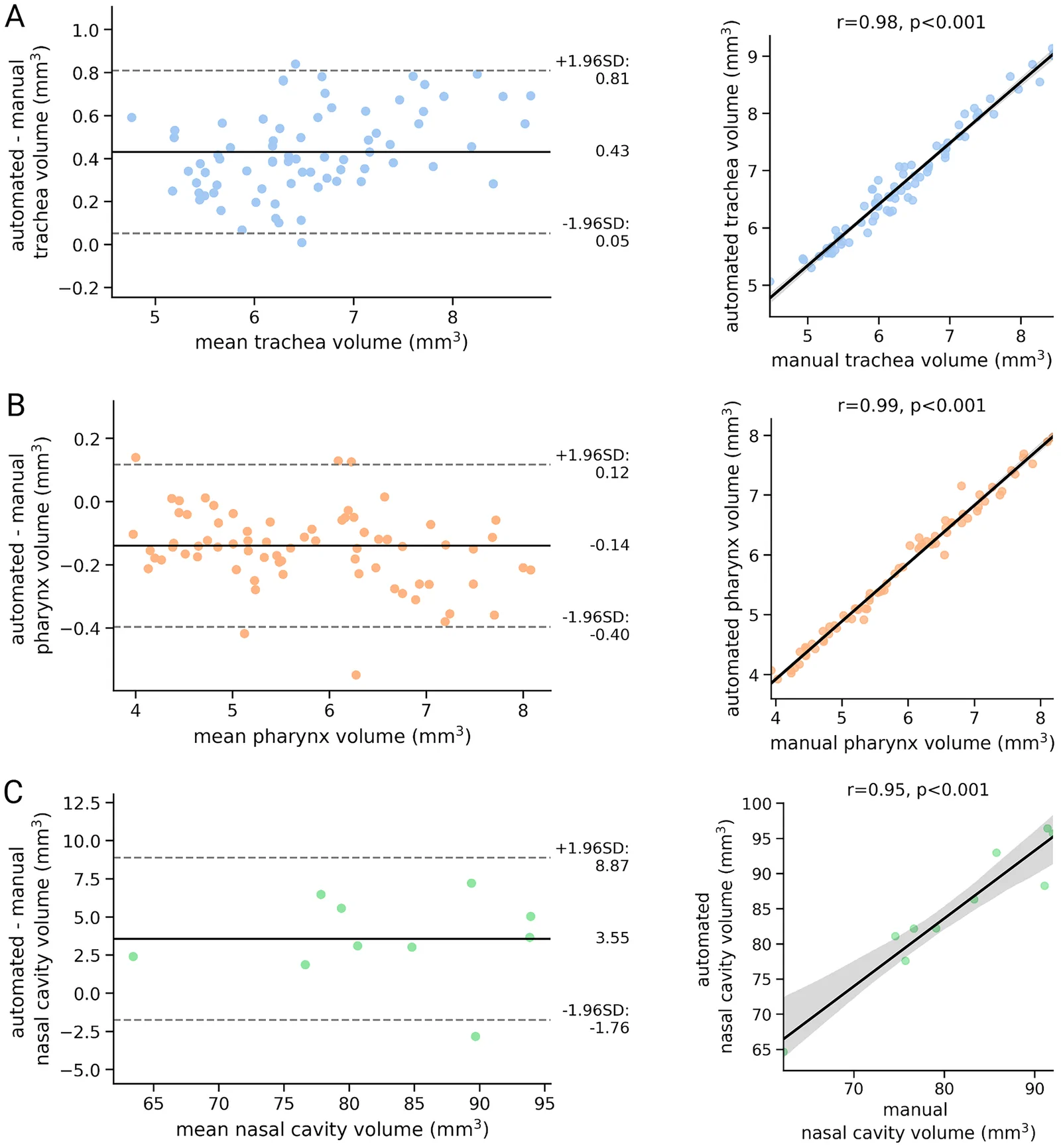
Agreement assessment between manual and automated measurements. Bland–Altman plots (left column) and scatter plots with linear regression model fit (right column) to assess agreement between manual and automated **(A)** trachea volume, **(B)** pharynx volume, **(C)** nasal cavity volume, on the external validation set. Pearson correlation coefficients are indicated on top of the scatter plots.

To further investigate whether the segmentation model could be used reliably for downstream biomarker quantification, we performed a detailed comparison of the results of volumetric biomarker extraction from the manually and automatically segmented upper airway. We generated Bland–Altman plots showing the mean difference and limits of agreement and assessed the Pearson correlation on the external validation set (Figure 4). The mean differences between manual and automated measurements were 0.43 mm^3^ for trachea volume, −0.14 mm^3^ for pharynx volume and 3.55 mm^3^ for nasal cavity volume, with nearly all datapoints lying within the limits of agreement. Strong correlations were observed between manual and automated measurements, as indicated by Pearson correlation coefficients of 0.98, 0.99, and 0.95 for the trachea, pharynx, and nasal cavity, respectively.

### 3.3 Biological relevance

**Figure 5:**
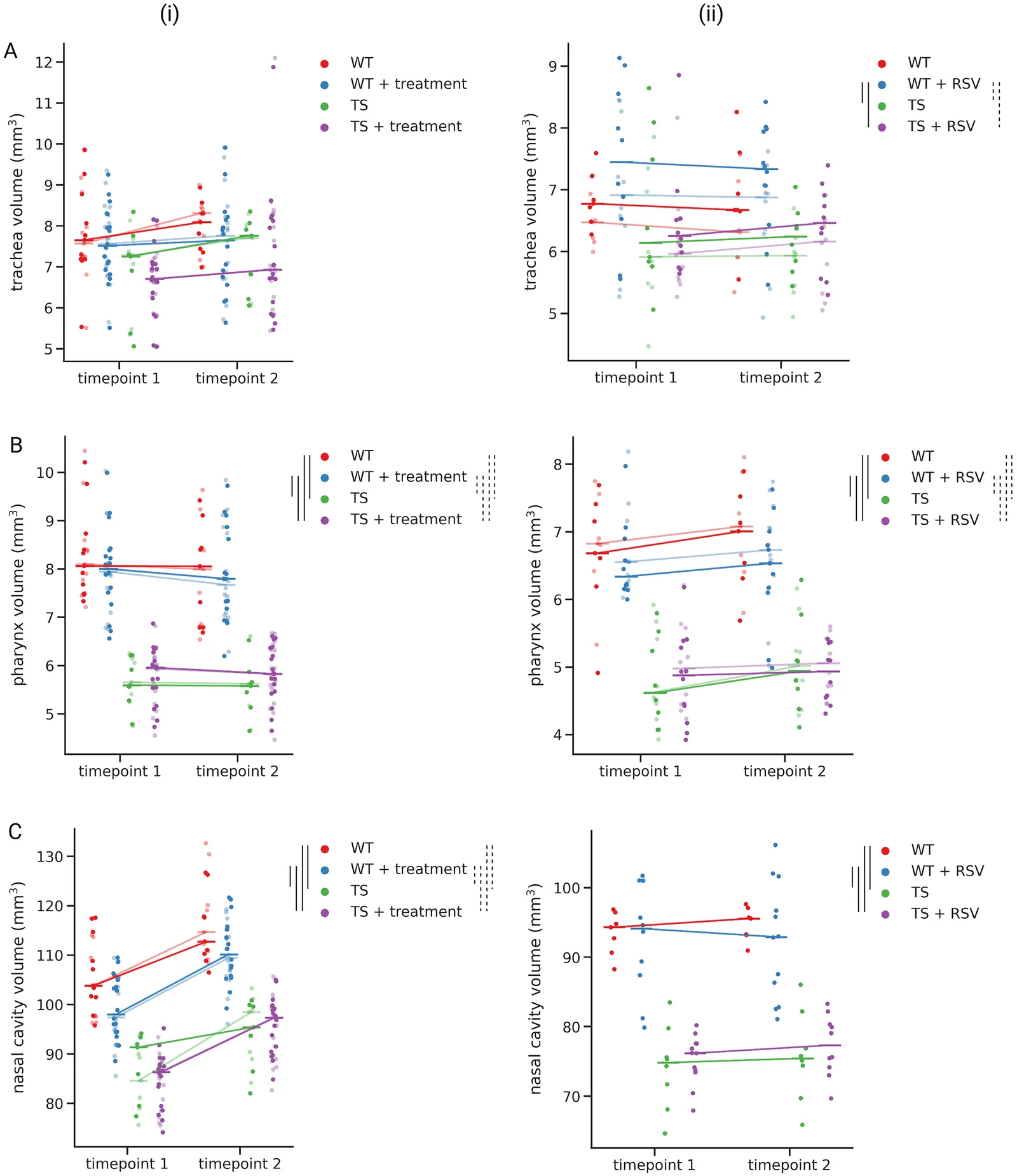
Comparison of automated vs. manual analysis of the upper airway in different experimental groups. **(A)** Trachea, **(B)** pharynx and **(C)** nasal cavity volumes quantified at two timepoints for (i) the internal validation set and (ii) external validation set. Automated volumes are shown as solid dots, with medians indicated by solid horizontal lines. Manual volumes are shown as transparent dots, with medians indicated by transparent horizontal lines. Statistical significance (alpha = 0.05) of *post-hoc* pairwise comparisons between groups, averaged across timepoints, is indicated by lines adjacent to the legend; solid for automated and dashed for manual results. In the external validation set, only automated measurements of nasal cavity were available for all datapoints.

To assess the utility and biological relevance of the developed DL–based automated pipeline, we examined its ability to identify meaningful anatomical differences in the upper airway of murine models. Specifically, we evaluated whether the automated pipeline reproduced the statistically significant differences in upper airway volumes between experimental groups in the internal and external validation sets, that were identified through manual analysis (Figure 5). All mixed-design ANOVA results are reported in Supplementary Tables S2 and S3. In the internal validation set, automated analysis replicated manual analysis results with TS groups showing smaller pharynx and nasal cavity volumes than WT groups. In the external validation set, automated analysis replicated manual analysis results with TS and TS + RSV showing smaller trachea volumes than WT + RSV, and TS groups showing smaller pharynx volumes than WT groups. Automated analysis also revealed smaller nasal cavity volumes for the TS groups compared to the WT groups, but this could not be compared with manual analysis as not all nasal cavities were manually segmented. Overall, these results suggest that the automated pipeline can detect the same differences as manual analysis.

## 4 Discussion

In this study, we presented a DL-based pipeline for automated 3D characterisation of the murine upper airway, including trachea, pharynx and nasal cavity, from micro-CT scans. A full-resolution 2D CNN based on the state-of-the-art nnU-Net framework was trained and internally evaluated in the axial plane using 5-fold cross-validation, on data from an experimental study involving WT and TS mice, with or without GTE-EGCG treatment. We evaluated the performance of our method on an independent experimental cohort investigating the effect of RSV infection in WT and TS mice, with scans acquired under slightly different imaging settings using the same scanner.

Our segmentation method was implemented using the nnU-Net framework, which was selected because it provides a standardized and extensively validated implementation of U-Net architectures with automatic adaptation of preprocessing, network configuration, training, and inference to the characteristics of the dataset. This minimizes manual hyperparameter tuning and facilitates reproducibility while providing a strong state-of-the-art baseline for biomedical image segmentation (28). We adopted an ensemble of 2D U-Nets rather than a 3D architecture because 2D models are considerably less demanding in terms of GPU memory, allowing training on full-resolution images with larger batch sizes which can improve optimization stability. In addition, their lower computational requirements make them more practical for deployment in research laboratories with limited hardware resources. Therefore, we consider our approach to provide an appropriate balance between segmentation accuracy, computational cost, and practical applicability.

To date, only two studies have performed a characterization of the murine upper airway using *in vivo* micro-CT: one examining eosinophilic upper airway inflammation, and another focusing on DS (15, 16). In both studies, a limited number of slices containing the upper airway were manually segmented prior to statistical analysis. However, this approach is time- and resource- intensive, prone to inter- and intra-observer variability and does not allow for full 3D characterization of the upper airway. To the best of our knowledge, we developed the first approach that enables fully automated segmentation of the murine upper airway on micro-CT, addressing the limitations of manual analysis and facilitating efficient analysis of large micro-CT datasets.

Our model showed consistently high performance across both internal cross-validation and external validation, for all subparts of the upper airway, although external validation of the nasal cavity was performed on a limited number of manually segmented scans and requires further validation on a larger dataset. We observed a small drop in performance for the trachea from internal to external validation, though the DSC remained above 0.9. Moreover, the various volumetric analyses revealed a systematic tendency toward slightly higher tracheal volumes in the automated segmentations compared with the manual reference standard in the external validation set. Further qualitative investigation showed that this performance drop is more likely attributable to subtle inconsistencies in the manual reference standard between the training and external validation set rather than reflecting a true model deficiency. Specifically, we found that the reference segmentation of the trachea started a few slices later in the external validation set compared to the training set, affecting the DSC and volumetric comparisons, and underscoring the inherent ambiguity in defining the tracheal bifurcate on micro-CT in practice. Our pipeline addresses this observer-related variability by delivering standardized and repeatable measurements.

More generally, although the DSC and RAVD demonstrated high overall segmentation performance, these global metrics do not fully reflect localized inaccuracies at anatomically challenging boundaries. Therefore, the reported performance metrics should be interpreted alongside qualitative assessment. In our study, local discrepancies were primarily confined to the tracheal bifurcation and the nasal cavity, where the intricate anatomy and heterogeneous tissue contrast make precise boundary delineation inherently challenging.

Among the different subparts of the upper airway, the nasal cavity is the most challenging to segment due to its complex anatomy and the heterogeneous contrast between its fine bony and soft-tissue structures. Consequently, manual delineation was highly time-consuming, with segmentation of a single nasal cavity requiring approximately 1 h on average. Due to this time-intensiveness, only 10 nasal cavities in the external validation set were manually segmented and used as independent reference standard for quantitative evaluation. For these same 10 scans, the automated segmentations were additionally visually inspected and manually corrected by the expert observer to assess the practical usability and the required correction effort of the automated segmentations. As these corrected segmentations were generated from the model output, they should not be interpreted as an independent reference standard or as a strict measure of model generalisability. Rather, the improved agreement between the automated and corrected segmentations suggests that, in many cases, only limited manual refinement of the automated segmentation was required. Nevertheless, the limited number of manually segmented nasal cavities in the external validation set warrants cautious interpretation of the external validation results for this structure. Despite these challenges, our results suggest that the proposed pipeline substantially reduces the manual workload associated with upper airway analysis and facilitates more standardized and efficient high-throughput analysis of large micro-CT datasets.

Importantly, our automated pipeline facilitates downstream extraction of airway biomarkers from the automated segmentations, including volumetric measurements, to quantify abnormalities of the upper airway. We demonstrated that upper airway volumes extracted from manual and automated segmentations are well in agreement, showing Pearson correlations close to 1 and small mean differences in the Bland–Altman analysis. The set of automatically extracted biomarkers could easily be extended with intensity-based measurements or volumetric measurements of subareas within the nasal cavity, for example applying thresholds on the histogram to extract the nasal mucosa area to study upper airway hyperresponsiveness in allergic rhinitis mouse models (15). In addition, our automated pipeline can provide the geometric inputs required for advanced analyses such as geometric morphometrics and, with further refinement, CFD, thereby opening new avenues for efficient study of variation in upper airway shape, airflow mechanics and resistance across experimental groups (4, 16).

Moreover, we created substantial evidence for the utility and biological relevance of our pipeline by showing that the automated measurements reproduced the statistically significant differences in upper airway volumes between experimental groups in the internal and external validation sets, that were originally identified through manual analysis. Consistent with previous findings in mice, we confirmed that TS mice present a smaller pharynx and nasal cavity than WT mice (16). Moreover, these preclinical findings align with clinical observations in individuals with DS, who typically present with reduced upper airway volumes (32).

Our work aligns with ongoing efforts to enhance the translational relevance of preclinical studies. By combining specialized small-animal imaging technologies with an automated quantification pipeline, we can extract direct, standardized measurements of anatomy and disease that can be compared with clinical imaging studies. This creates a direct bridge between mouse and human airway assessment, enabling cross-species comparisons that can refine preclinical hypotheses and guide therapeutic development.

Despite the following limitations, our study opens new avenues for respiratory research using preclinical mouse models. First, only a limited number of independently manually segmented nasal cavities were available in the external validation set. Consequently, external validation for the nasal cavity was performed on a relatively small subset of scans. Larger external datasets with independently generated annotations and blinded multi-observer evaluation would further strengthen assessment of model generalisability for the nasal cavity. Second, although the reference segmentations were generated by experienced annotators following a standardized annotation procedure, formal inter- and intra-observer variability analyses were not performed. Consequently, the reproducibility of the manual reference standard could not be quantified directly. Future studies could include dedicated observer variability assessments to further characterize annotation uncertainty and provide additional context for the performance of automated segmentation methods. Third, all micro-CT scans used in this study were acquired on the same scanner. Further validation on multicenter datasets including scans from other scanners is warranted to expand the generalizability of the model'. However, we implemented an image standardization approach using HU scaling which minimizes scanner-induced differences. Fourth, extended validation in other preclinical disease models such as allergic rhinitis could usefully demonstrate the wide applicability of the pipeline across diverse airway-related research lines. Lastly, to enable advanced CFD analyses, the automated segmentation of the nasal cavity requires further refinement to isolate the air spaces and exclude the inner cartilage. This could be achieved through the application of intensity thresholding within the automated segmentation, in combination with manual refinement.

In conclusion, we developed and validated a DL-based pipeline that enables fully automated and reproducible segmentation and quantification of the murine upper airway from micro-CT, demonstrating robust performance across diverse mouse genotypes (WT and TS), experimental conditions (treatment and RSV infection) and imaging protocols. By substantially reducing manual workload and enhancing measurement consistency, this tool facilitates in-depth, mechanistically driven studies of upper airway anatomy and obstruction in preclinical respiratory research, a domain that remains largely understudied. It also provides a foundation for advanced morphometric and, with further refinement, CFD analyses and strengthens the translational bridge between experimental and clinical research.

## Data Availability

The raw data supporting the conclusions of this article will be made available by the authors, without undue reservation.
