# Supplementary material for: An automated deep learning-based pipeline for 3D characterisation of the murine upper airway from micro-CT
Source: Front Med Technol. 2026 Aug 11;8:1823048. doi: 10.3389/fmedt.2026.1823048 (PMC13503376; doi:10.3389/fmedt.2026.1823048)
Supplement: Supplementary file 1 [file Supplementaryfile1.docx]

***Supplementary Material***

1 **Supplementary tables**

**Table S1** Summary of the distribution of animals and scans across the five folds from five-fold cross-validation. The table includes the number of animals and scans per fold, as well as the distribution of sex, genotype and treatment group.

| Fold | Nr of animals | Nr of scans | Male, n (%) | Trisomic, n (%) | Treated, n (%) |
| --- | --- | --- | --- | --- | --- |
| **1** | 11 | 21 | 4 (36) | 5 (45) | 7 (64) |
| **2** | 11 | 22 | 4 (36) | 5 (45) | 7 (64) |
| **3** | 11 | 21 | 5 (45) | 5 (45) | 7 (64) |
| **4** | 11 | 21 | 4 (36) | 5 (45) | 8 (73) |
| **5** | 11 | 21 | 5 (45) | 5 (45) | 7 (64) |

Table S2 Mixed-design ANOVA results for upper airway volumes in the internal validation set, with experimental group as the between-subjects factor and time as the within-subjects factor, for both manual and automated analyses.

| **Region** | **Method** | **Effect** | **F (df1, df2)** | **p-value** |
| --- | --- | --- | --- | --- |
| Trachea | Manual | Group | 1.74 (3, 46) | 0.17 |
|  |  | Time | 12.56 (1, 46) | **0.00092** |
|  |  | Interaction | 1.76 (3, 46) | 0.17 |
|  | Automated | Group | 1.73 (3, 46) | 0.17 |
|  |  | Time | 7.40 (1, 46) | **0.0092** |
|  |  | Interaction | 1.52 (3, 46) | 0.22 |
| Pharynx | Manual | Group | 36.16 (3, 46) | **<0.0001** |
|  |  | Time | 1.48 (1, 46) | 0.23 |
|  |  | Interaction | 1.45 (3, 46) | 0.24 |
|  | Automated | Group | 35.98 (3, 46) | **<0.0001** |
|  |  | Time | 3.46 (1, 46) | 0.069 |
|  |  | Interaction | 1.62 (3, 46) | 0.20 |
| Nasal cavity | Manual | Group | 28.89 (3, 46) | **<0.0001** |
|  |  | Time | 585.61 (1, 46) | **<0.0001** |
|  |  | Interaction | 1.42 (3, 46) | 0.25 |
|  | Automated | Group | 30.30 (3, 46) | **<0.0001** |
|  |  | Time | 1204.32 (1, 46) | **<0.0001** |
|  |  | Interaction | 17.71 (3, 46) | **<0.0001** |

Table S3 Mixed-design ANOVA results for upper airway volumes in the external validation set, with experimental group as the between-subjects factor and time as the within-subjects factor, for both manual and automated analyses.

| **Region** | **Method** | **Effect** | **F (df1, df2)** | **p-value** |
| --- | --- | --- | --- | --- |
| Trachea | Manual | Group | 3.20 (3, 31) | **0.037** |
|  |  | Time | 2.05 (1, 31) | 0.16 |
|  |  | Interaction | 0.15 (3, 31) | 0.93 |
|  | Automated | Group | 3.32 (3, 31) | **0.032** |
|  |  | Time | 0.90 (1, 31) | 0.35 |
|  |  | Interaction | 0.38 (3, 31) | 0.77 |
| Pharynx | Manual | Group | 25.51 (3, 31) | **<0.0001** |
|  |  | Time | 3.48 (1, 31) | 0.071 |
|  |  | Interaction | 0.17 (3, 31) | 0.92 |
|  | Automated | Group | 25.29 (3, 31) | **<0.0001** |
|  |  | Time | 3.07 (1, 31) | 0.090 |
|  |  | Interaction | 0.27 (3, 31) | 0.84 |
| Nasal cavity | Manual | Group | / | / |
|  |  | Time | / | / |
|  |  | Interaction | / | / |
|  | Automated | Group | 28.55 (3, 31) | **<0.0001** |
|  |  | Time | 26.05 (1, 31) | **<0.0001** |
|  |  | Interaction | 0.07 (3, 31) | 0.98 |

2 **Supplementary figures**

Figure S1 Schematic overview of the entire development and evaluation process, including image acquisition and reconstruction, manual annotation, preprocessing, model training and inference, post-processing, biomarker extraction, and statistical evaluation.
